# Accurately identifying hemagglutinin using sequence information and machine learning methods

**DOI:** 10.3389/fmed.2023.1281880

**Published:** 2023-10-31

**Authors:** Xidan Zou, Liping Ren, Peiling Cai, Yang Zhang, Hui Ding, Kejun Deng, Xiaolong Yu, Hao Lin, Chengbing Huang

**Affiliations:** ^1^School of Life Science and Technology, Center for Informational Biology, University of Electronic Science and Technology of China, Chengdu, China; ^2^School of Healthcare Technology, Chengdu Neusoft University, Chengdu, China; ^3^School of Basic Medical Sciences, Chengdu University, Chengdu, China; ^4^Innovative Institute of Chinese Medicine and Pharmacy, Academy for Interdiscipline, Chengdu University of Traditional Chinese Medicine, Chengdu, China; ^5^School of Materials Science and Engineering, Hainan University, Haikou, China; ^6^School of Computer Science and Technology, Aba Teachers University, Aba, China

**Keywords:** hemagglutinin, machine learning, sequence features, feature extraction, stacking

## Abstract

**Introduction:**

Hemagglutinin (HA) is responsible for facilitating viral entry and infection by promoting the fusion between the host membrane and the virus. Given its significance in the process of influenza virus infestation, HA has garnered attention as a target for influenza drug and vaccine development. Thus, accurately identifying HA is crucial for the development of targeted vaccine drugs. However, the identification of HA using in-silico methods is still lacking. This study aims to design a computational model to identify HA.

**Methods:**

In this study, a benchmark dataset comprising 106 HA and 106 non-HA sequences were obtained from UniProt. Various sequence-based features were used to formulate samples. By perform feature optimization and inputting them four kinds of machine learning methods, we constructed an integrated classifier model using the stacking algorithm.

**Results and discussion:**

The model achieved an accuracy of 95.85% and with an area under the receiver operating characteristic (ROC) curve of 0.9863 in the 5-fold cross-validation. In the independent test, the model exhibited an accuracy of 93.18% and with an area under the ROC curve of 0.9793. The code can be found from https://github.com/Zouxidan/HA_predict.git. The proposed model has excellent prediction performance. The model will provide convenience for biochemical scholars for the study of HA.

## 1. Introduction

Influenza is a contagious respiratory disease, posing a significant threat to human health and causing varying degrees of disease burden globally (1, 2). Hemagglutinin (HA), a glycoprotein on the surface of influenza viruses, mediates viral entry and infection by binding to host sialic acid receptors (3). The highly conserved stem or stalk region of HA has been identified as a promising target for the development of a universal influenza vaccine (4). Accurate identification of HA is crucial for targeted vaccine and drug development.

With the increasing maturity of protein sequence coding methods and machine learning algorithms, sequence-based protein recognition has been an effective approach for rapid identification of protein. It achieves classification and identification of specific proteins using protein sequence coding methods and machine learning algorithms, which has been widely used in the prediction studies of cell-penetrating peptides (5), hemolytic peptide (6), anti-cancer peptides (7), hormone proteins (8), autophagy proteins (9), and Anti-CRISPR proteins (10), etc., because of its high recognition accuracy in the protein identification study.

Despite the pivotal role of HA in influenza virus infection, existing machine learning-based research on HA has primarily focused on influenza virus subtype classification (11, 12), influenza virus host prediction (13), influenza virus mutation and evolution prediction (14), HA structure–function analysis (15), and influenza virus pathogenicity and prevalence prediction (16). However, there are currently no approaches for HA identification based on HA sequence information and machine learning techniques.

**Figure 1:**
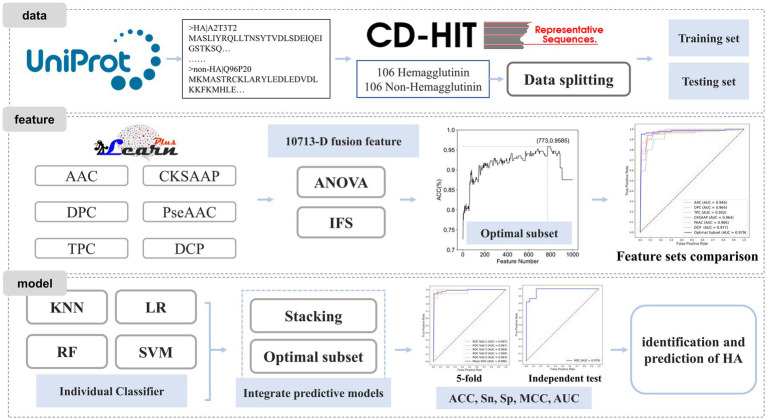
Workflow diagram for constructing the HA prediction model.

In this study, we proposed a machine learning-based prediction model for HA to achieve effective identification. Firstly, we constructed a benchmark dataset based on existing protein databases. Next, we employed feature extraction methods to encode the protein sequences. Subsequently, we fused all the extracted features and utilized the analysis of variance (ANOVA) combined with incremental feature selection (IFS) strategies to obtain the most informative feature subset. Finally, the HA prediction model was developed based on this optimal feature subset. The workflow is shown in Figure 1.

## 2. Method and materials

### 2.1. Benchmark dataset

A benchmark dataset is essential for bioinformatics analysis (17, 18). The dataset used in this study was collected from the Universal Protein Resource (UniProt) (19). To ensure the quality of the dataset, several pre-processing steps were performed. Protein sequences containing nonstandard letters (e.g., ‘B’, ‘U’, ‘X’, ‘Z’) were eliminated. Redundancy removal was done using CD-HIT (20) to remove sequences with high similarity. The cutoff value was set to 80%, and sequences with a similarity higher than 80% were removed. The non-HA dataset was down-sampled to ensure a balanced dataset with equal positive and negative samples. The final benchmark dataset consisted of 212 protein sequences, including 106 HA and 106 non-HA samples. The dataset was randomly split into a training dataset and a test dataset in a 4:1 ratio. The above-mentioned model training set data and test set data are included in https://github.com/Zouxidan/HA_predict.git. At the same time, a dataset named ‘predict_data.txt’ for testing is also included.

### 2.2. Feature extraction

Feature extraction plays a crucial role in protein identification and prediction (10, 21–25). However, machine learning algorithms cannot directly process protein sequence information for computation and model construction. Therefore, it is necessary to convert protein sequence information into numerical data that can be understood and utilized by machine learning algorithms (26–29). Here, we employed various methods for feature extraction of protein sequences, including Amino Acid Composition (AAC), Dipeptide Composition (DPC), Tripeptide Composition (TPC), Composition of k-spaced Amino Acid Pairs (CKSAAP), Pseudo-Amino Acid Composition (PseAAC), PseAAC of Distance-Pairs and Reduced Alphabet (DCP). These sequence feature extraction approaches have been widely adopted in the field of bioinformatics (30–32). The implementation of these feature extraction methods was based on iLearnPlus (33).

A protein sequence *P* of length *L* can be represented as:


(1) $$P=R_{1}R_{2}R_{3}R_{4}R_{5}R_{6}\cdots R_{L}$$

where *R*_1_ denotes the first amino acid of the sequence, *R*_2_ denotes the second amino acid, and so on.

#### 2.2.1. AAC

AAC is a commonly used method for protein sequence feature extraction, which involves 20 feature vectors. AAC was defined as:


(2) $$f_{i}=\frac{N\left(a_{i}\right)}{\sum_{i}^{20}N\left(a_{i}\right)}=\frac{N\left(a_{i}\right)}{L}$$

where *a_i_* denotes the *i*-th natural amino acid and *N*(*a_i_*) denotes the frequency of amino acid *a_i_* in the protein sequence.

#### 2.2.2. DPC

Similar to AAC, DPC counts the frequency of amino acids, but it focuses on the frequency of two adjacent amino acids in a protein sequence. DPC was defined as:


(3) $$f_{i,j}=\frac{N\left(a_{i},a_{j}\right)}{\sum_{i}^{20}\sum_{j}^{20}N\left(a_{i},a_{j}\right)}=\frac{N\left(a_{i},a_{j}\right)}{L-1}$$

where (*a_i_*, *a_j_*) denotes two adjacent amino acids and *N*(*a_i_*, *a_j_*) denotes the frequency of the amino acid pair (*a_i_*, *a_j_*) in the protein sequence.

#### 2.2.3. TPC

TPC is another feature extraction method that considers the relationship among three adjacent amino acids, providing more protein sequence information compared to AAC and DPC. TPC was defined as:


(4) $$f_{i,j,z}=\frac{N\left(a_{i},a_{j},a_{z}\right)}{\sum_{i}^{20}\sum_{j}^{20}\sum_{z}^{20}N\left(a_{i},a_{j},a_{z}\right)}=\frac{N\left(a_{i},a_{j},a_{z}\right)}{L-2}$$

where (*a_i_*, *a_j_*, *a_z_*) denotes the combination of three adjacent amino acids, and *N*(*a_i_*, *a_j_*, *a_z_*) denotes the frequency of the tripeptide combination (*a_i_*, *a_j_*, *a_z_*) in the protein sequence.

#### 2.2.4. CKSAAP

To obtain further sequence information, Chen et al. proposed CKSAAP (34) which was defined as:


(5) $$f_{i,j,k}=\frac{N\left(a_{i},x_{k},a_{z}\right)}{\sum_{i}^{20}\sum_{j}^{20}N\left(a_{i},x_{k},a_{z}\right)}=\frac{N\left(a_{i},x_{k},a_{z}\right)}{L-k-1}$$

where *k* denotes the number of amino acids spaced between two amino acids, *x_k_* denotes *k* arbitrary amino acids, (*a_i_*, *x_k_*, *a_j_*) denotes the spaced amino acid pair, and *N*(*a_i_*, *x_k_*, *a_j_*) denotes the frequency of the spaced amino acid pair (*a_i_*, *x_k_*, *a_j_*) in the protein sequence.

#### 2.2.5. PseAAC

To incorporate protein sequence ordinal information and improve prediction quality, a powerful feature, called PseAAC, was proposed, which incorporated the physicochemical characteristics of amino acids. PseAAC was defined as:


(6) $$f_{i}=\{\begin{array}{c} \frac{x_{i}}{\sum_{i=1}^{20}x_{i}+\omega \sum_{j=1}^{\lambda }\theta_{j}}\;\left(0<i\leq 20\right)\\ \frac{\omega \theta_{i-20}}{\sum_{i=1}^{20}x_{i}+\omega \sum_{j=1}^{\lambda }\theta_{j}}\;\left(20<i\leq 20+\lambda \right) \end{array}$$

where *x_i_* denotes the normalized amino acid frequency, *ω* denotes the weight factor for short-range and long-range, and *θ_j_* denotes the *j*-th sequence correlation factor.

*θ_j_* was calculated as:


(7) $$\theta_{j}=\frac{1}{L-j}\overset{L-j}{\underset{i=1}{\sum }}\Theta \left(R_{i}+R_{i+j}\right)$$

*Θ*(*R_i_* + *R_i + j_*) was defined as:


(8) $$\begin{array}{l} \Theta \left(R_{i}+R_{i+j}\right)=\\ \frac{{\left[H_{1}\left(R_{i+j}\right)-H_{1}\left(R_{i}\right)\right]}^{2}+{\left[H_{2}\left(R_{i+j}\right)-H_{2}\left(R_{i}\right)\right]}^{2}+{\left[M\left(R_{i+j}\right)-M\left(R_{i}\right)\right]}^{2}}{3} \end{array}$$

where *H*_1_(*R_i_*), *H*_2_(*R_i_*), and *M*(*R_i_*) denote the standardized hydrophobicity, standardized hydrophilicity, and standardized side chain mass of the amino acid *R_i_*, respectively.

The hydrophobicity, hydrophilicity, and side chain mass of amino acids were standardized using the following equations:


(9) $$\left\{ \begin{array}{c} H_{1}\left(R_{i}\right)=\frac{H_{1}^{0}\left(R_{i}\right)-\sum_{i=1}^{20}\frac{H_{1}^{0}\left(R_{i}\right)}{20}}{\sigma \left(H_{1}^{0}\right)}\\ H_{2}\left(R_{i}\right)=\frac{H_{2}^{0}\left(R_{i}\right)-\sum_{i=1}^{20}\frac{H_{2}^{0}\left(R_{i}\right)}{20}}{\sigma \left(H_{2}^{0}\right)}\\ M\left(R_{i}\right)=\frac{M^{0}\left(R_{i}\right)-\sum_{i=1}^{20}\frac{M^{0}\left(R_{i}\right)}{20}}{\sigma \left(M^{0}\right)} \end{array}\right.$$

where *H*_1_(*R_i_*), *H*_2_(*R_i_*), and *M*(*R_i_*) denote the standardized hydrophobicity, standardized hydrophilicity, and standardized side chain mass of amino acids, respectively, and $H_{1}^{0}\left(R_{i}\right)$, $H_{2}^{0}\left(R_{i}\right)$, and $M^{0}\left(R_{i}\right)$ denote the corresponding raw physicochemical properties of amino acids.

#### 2.2.6. DCP

To incorporate more protein sequence order information and reduce the impact of high-dimensional features, Liu et al. proposed DCP (35). Based on a validated amino acid simplification alphabet scheme (36), three simplified amino acid alphabets were defined as:


(10) $$\left\{ \begin{array}{c} cp\left(13\right)=\left\{\begin{array}{l} MF;\;IL;\;V;\;A;\;C;\;\mathrm{WYQHP};\;G;\;T;\;S;\;N;\;\\ RK;\;D;\;E \end{array}\right\}\\ cp\left(14\right)=\left\{\begin{array}{l} IMV;\;L;\;F;\;WY;\;G;\;P;\;C;\;A;\;S;\;T;\;N;\;\\ \mathrm{HRKQ};\;E;\;D \end{array}\right\}\\ cp\left(19\right)=\left\{\begin{array}{l} P;\;G;\;E;\;K;\;R;\;Q;\;D;\;S;\;N;\;T;\;H;\;C;\;I;\\ V;\;W;\;YF;\;A;\;L;\;M \end{array}\right\} \end{array}\right.$$

For any simplified amino acid alphabet, DCP was defined as:


(11) $$f_{i,j,z}=\frac{N_{d}\left(cp_{i},cp_{j}\right)}{\sum_{i}^{z}\sum_{j}^{z}N_{d}\left(cp_{i},cp_{j}\right)}$$

where *z* denotes the number of amino acid clusters in the simplified alphabet, and $N_{d}\left(cp_{i},cp_{j}\right)$ denotes the frequency of any two amino acid clusters with distance d in the protein sequence.

In this study, the following parameters were used for protein sequence feature extraction: *k* = 1 for CKSAAP (amino acid spacing value), *λ* = 10 for PseAAC (number of amino acid theoretical properties), and *ω* = 0.7 for the weight factor for short-range and long-range. Consequently, we extracted features that include 20-dimensional AAC, 400-dimensional DPC, 8000-dimensional TPC, 800-dimensional CKSAAP, 30-dimensional PseAAC, and 1,463-dimensional DCP.

### 2.3. Feature fusion and selection

Different feature extraction methods offer diverse interpretations and representations of protein sequences. Relying solely on a single feature extraction method may limit the information provided by a single feature. To obtain a more comprehensive and reliable interpretation of protein sequences, we fused all features to create a fused feature set, resulting in a 10,713-dimensional feature set (20 + 400 + 8,000 + 800 + 30 + 1,463). We then selected the optimal feature subset using ANOVA and IFS.

ANOVA, a widely used feature selection tool, tests the difference in means between groups to determine whether the independent variable influences the dependent variable. Its high accuracy has made it an effective choice for feature selection (8). For a feature *f*, its *F*-value was calculated based on the principle of ANOVA as follows:


(12) $$F\left(f\right)=\frac{SSA/\left(K-1\right)}{SSE/\left(N-K\right)}$$

where *F*(*f*) represents the *F*-value of feature *f*, *SSA* represents the sum of squares between groups, *SSE* represents the sum of squares within groups, *K*-1 and *N-K* denote the degrees of freedom between and within groups, respectively. *N* is the total number of samples, and *K* is the number of groups.

*SSA* and *SSE* were calculated as follows:


(13) $$\left\{ \begin{array}{c} SSA=\overset{K}{\underset{i=1}{\sum }}\overset{k_{i}}{\underset{j=1}{\sum }}{\left(f\left(i,j\right)-\frac{\sum_{j=1}^{k_{i}}f\left(i,j\right)}{k_{i}}\right)}^{2}\\ SSE=\overset{K}{\underset{i=1}{\sum }}k_{i}{\left(\frac{\sum_{j=1}^{k_{i}}f\left(i,j\right)}{k_{i}}-\frac{\sum_{i=1}^{K}\sum_{j=1}^{k_{i}}f\left(i,j\right)}{\sum_{i=1}^{K}k_{i}}\right)}^{2} \end{array}\right.$$

where $f\left(i,j\right)$ denotes the *j*-th feature of the *i*-th group, *K* represents the number of groups, and *k_i_* represents the total number of samples in the *i*-th group.

A larger *F*-value indicates a stronger influence of the feature on data classification, thereby contributing more to the data classification results. In the feature set, the large amount of data, redundant data and noise will not only result in higher computational costs, but also cause the phenomenon of overfitting or reduced accuracy of the prediction model. The above fusion feature set contains 10,713 features, which is a large number of features. For saving computational time and reducing computational cost, we firstly use ANOVA to initially filter to obtain the 1,000 features which have the greatest influence on the classification results.

Next, the optimal subset of features was determined by searching the top 1,000 features ranked by *F*-value using IFS. IFS is a frequently employed feature selection method in the field of bioinformatics (37, 38). The specific process of IFS is as follows. Firstly, all features were sorted in descending order according to their *F*-values obtained from ANOVA. Then, each feature was sequentially added to the feature set, and a model was constructed using support vector machine (SVM) for each newly formed feature subset. Grid search was utilized to obtain optimal models, and their performance was evaluated using 5-fold cross-validation. The optimal feature subset was defined as the set of features that maximized the model’s accuracy.

### 2.4. Machine learning methodology and modeling

The advancement of machine learning has provided an effective approach to solving biological problems (39–42). Utilizing machine learning techniques to identify proteins based on sequence features has proven to be a rapid and widely applied method in various studies (43–45).

Constructing appropriate models is crucial for achieving accurate and robust predictions. In this study, we selected four commonly used machine learning algorithms, namely K-nearest neighbor (KNN) (46), logistic regression (LR) (47), random forest (RF) (48), and SVM (49), to build the fundamental classifier model for the HA dataset. The optimal parameters for each algorithm were obtained using grid search. To further enhance the model’s accuracy and generalization ability, we developed an integrated classifier model by combining the four basic classifier models. The Stacking algorithm was employed, with logistic regression serving as the second-layer classifier. All the machine learning models utilized in this study were implemented using scikit-learn (50).

KNN is a simple yet effective machine learning algorithm based on the implementation of the distance between data and data. LR is a binary classification algorithm based on the sigmoid function, which classifies samples by their corresponding output values. In RF, the result of prediction is determined by the vote or average of decision trees. The basic principle of SVM is to separate two classes of training data by defining a hyperplane and maximizing the distance between the two classes.

The Stacking algorithm is one of the widely used integrated learning methods, which obtains predictive models with higher accuracy and better generalization ability by combining basic classifier models. The Stacking algorithm was initially proposed by Wolpert (51). Its basic idea is to obtain an optimal integrated classifier model by training and combining multiple basic classifier models. In the Stacking algorithm, machine learning algorithms with strong learning and fitting capabilities are frequently used to construct basic classifier models for adequate learning and interpretation of training data. To reduce the degree of overfitting, simple algorithms with strong interpretations are commonly used to construct integrated classifier models.

### 2.5. Performance evaluation

To assess the effectiveness of the constructed models, we employed 5-fold cross-validation and independent testing. The performance of the proposed model was evaluated using several metrics, including accuracy (*ACC*), sensitivity (*Sn*), specificity (*Sp*), Matthew’s correlation coefficient (*MCC*), and the area under the receiver operating characteristic curve (*AUC*) (27, 52–56). *ACC*, *Sn*, *Sp*, and *MCC* were expressed as:


(14) $$ACC=\frac{TP+TN}{TP+TN+FP+FN}$$

(15) $$Sn=\frac{TP}{TP+FN}$$

(16) $$Sp=\frac{TN}{TN+FP}$$

(17) $$MCC=\frac{TP\times TN-FP\times FN}{\sqrt{\left(TP+FN\right)\left(TP+FP\right)\left(TN+FP\right)\left(TN+FN\right)}}$$

where *TP*, *TN*, *FP*, and *FN* represent the following respectively: correctly identified positive samples, correctly identified negative samples, incorrectly identified negative samples, and incorrectly identified positive samples.

Additionally, we utilized the receiver operating characteristic (ROC) curve to evaluate model performance. A higher *AUC* value indicates better model performance, as it reflects the proximity to 1 according to the underlying principle.

## 3. Results and discussion

### 3.1. Optimal feature subset

**Figure 2:**
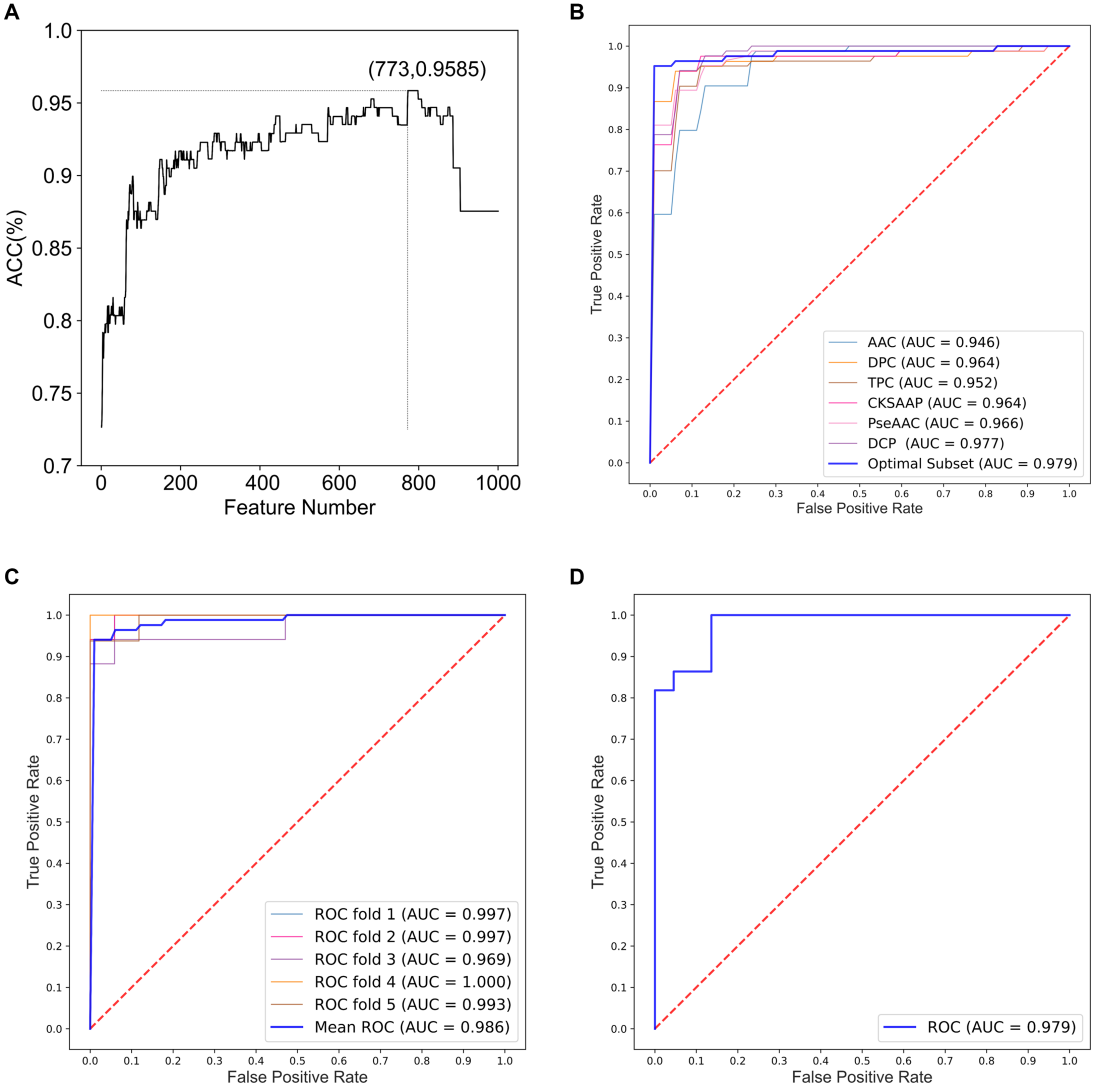
Performance analysis for optimal feature subsets and HA prediction models. **(A)** IFS curve for fusion features. **(B)** ROC curves of models constructed based on optimal feature subsets and six single feature sets. **(C)** ROC curves of the integrated classifier model with 5-fold cross-validation. **(D)** ROC curves of the integrated classifier model with independent testing.

We constructed optimal feature subsets using ANOVA and IFS and evaluated the models for each subset using the *ACC*. Figure 2A shows the IFS curve for the fusion feature set. When the feature set contained 773 features, the prediction model achieved a maximum *ACC* value of 0.9585.

In optimal feature subset, 13-dimensional AAC, 73-dimensional DPC, 629-dimensional TPC, 29-dimensional CKSAAP, and 29-dimensional DCP features are included. Notably, PseAAC is not included in this subset, suggesting that it is less effective in classifying HA compared to the other features. Furthermore, TPC has the highest proportion in optimal feature subset, indicating that TPC provides the best identification and differentiation ability among the six methods for feature extraction.

To demonstrate the impact of optimal feature subsets on model performance, we compared the performance of SVM prediction models constructed with optimal feature subsets to those constructed with six single feature sets. Each model was optimized using grid search within the same parameter range, and all models were evaluated using 5-fold cross-validation. Table 1 presents the results of the comparison, and Figure 2B shows the ROC curves for the 5-fold cross-validation of these models. The model constructed with the optimal feature subset achieved an *ACC* of 94.06% and an *AUC* of 0.970, outperforming the models constructed with other single feature sets. These results indicate that the optimal feature subset significantly improved the model’s prediction performance.

**Table 1:** Performance of models constructed based on optimal feature subsets and six single feature sets.

Feature type	*Sn*(%)	*Sp*(%)	*MCC*	*ACC*(%)	*AUC*
AAC	86.99	84.56	0.7324	85.74	0.9463
DPC	93.97	91.62	0.8597	92.83	0.9643
TPC	92.87	91.62	0.8512	92.30	0.9773
CKSAAP	92.79	89.26	0.8296	91.07	0.9636
PseAAC	85.66	92.79	0.7941	89.29	0.9518
DCP	86.99	89.26	0.7650	88.13	0.9658
Optimal subset	94.04	94.04	0.8825	94.06	0.9790

### 3.2. Model construction and evaluation

We constructed four basic models and an integrated model based on the optimal feature subsets. The optimal parameters for each algorithm were as follows: *K* = 52 for KNN, *n* = 62 for RF, *f* = 6 for the number of features considered during best-split search, *ξ* = 4 for the SVM kernel parameter, and *C* = 32 for the regularization parameter.

Table 2 presents the performance comparison of different classifier models using two testing methods. Figures 2C,D show the ROC curve of the constructed integration model using these wo testing methods. With 5-fold cross-validation, the proposed integrated model achieved an *ACC* of 95.85% and an *AUC* of 0.9863. On the independent test set, the integrated model achieved an *ACC* of 93.18% and an *AUC* of 0.9793. These results demonstrate that the proposed integrated model exhibited better HA prediction capability, improved model performance, and enhanced generalization ability compared to a single model.

**Table 2:** Performance of the integrated classifier model and the four basic classifier models.

category	Classifiers	*Sn*(%)	*Sp*(%)	*MCC*	*ACC*(%)	*AUC*
5-fold	KNN	91.69	83.38	0.7603	87.49	0.9250
	LR	91.69	72.57	0.6629	82.09	0.9266
	RF	95.29	89.34	0.8482	92.30	0.9645
	SVM	94.04	96.47	0.9070	95.26	0.9790
	Stacking	95.22	96.47	0.9179	95.85	0.9863
Independent test	KNN	90.91	86.36	0.7735	88.64	0.9483
	LR	95.45	81.82	0.7800	88.64	0.9566
	RF	90.91	90.91	0.8182	90.91	0.9793
	SVM	100.00	81.82	0.8321	90.91	0.9752
	Stacking	100.00	86.36	0.8718	93.18	0.9793

### 3.3. Comparison of other machine learning algorithms

We have created two models based on optimal feature subsets and compared their performance to demonstrate the superiority of our proposed model. The comparison results are presented in Table 3, where we compared the model constructed with the XGboost algorithm with our proposed model. The main parameters of the model constructed based on the XGboost algorithm are as follows: *max_depth* = 3, *learning_rate* = 0.16, *colsample_bytree* = 0.85, *subsample* = 0.75. The results in Table 3 show that our model has good classification performance.

**Table 3:** Performance of the stacking classifier model and the XGboost classifier models.

category	Classifiers	*Sn*(%)	*Sp*(%)	*MCC*	*ACC*(%)	*AUC*
5-fold	XGboost	92.94	88.01	0.8188	90.48	0.9675
	Stacking	95.22	96.47	0.9179	95.85	0.9863
Independent test	XGboost	100.00	81.82	0.8321	90.91	0.9917
	Stacking	100.00	86.36	0.8718	93.18	0.9793

### 3.4. Leave-one-out validation of the model

Due to the small sample data size, model robustness may be questioned. To ensure credible results, we use the leave-one-out method to re-validate model performance. The results of the model performance evaluation based on the leave-one-out method are shown in Table 4. In the performance evaluation of the model using the leave-one-out method, the model achieves an *ACC* of 93.45% and an *AUC* of 0.9846. The model shows good performance on both cross-validation methods, signifying its stability and the reliability of its classification outcomes.

**Table 4:** Performance evaluation based on the leave-one-out method.

category	Classifiers	*Sn*(%)	*Sp*(%)	*MCC*	*ACC*(%)	*AUC*
Leave-one-out	Stacking	92.86	94.05	0.8691	93.45	0.9846

## 4. Conclusion

Hemagglutinin (HA) is a vital glycoprotein found on the surface of influenza viruses, and accurately identifying HA is crucial for the development of targeted vaccine drugs. In this study, we proposed a prediction model based on HA protein sequence features. The model was constructed using the Stacking algorithm, incorporating an optimal subset of features and a basic classifier model. Our results demonstrated that the constructed model exhibits excellent predictive capacity and generalization ability.

We anticipate that the model will prove valuable in the effective identification and prediction of HA. Moving forward, we plan to explore additional feature extraction methods and optimize our prediction model to further enhance its performance. Additionally, we are committed to developing an accessible web server to facilitate the identification and prediction of HA.

In summary, our research provides a promising approach to accurately identifying HA and lays the foundation for the development of targeted vaccine drugs. We believe that our findings contribute to the advancement of influenza research and offer valuable insights for future studies in this field.

## Data availability statement

The original contributions presented in the study are included in the article/supplementary material, further inquiries can be directed to the corresponding authors.

## Author contributions

XZ: Data curation, Formal analysis, Methodology, Visualization, Writing – original draft. LR: Formal analysis, Investigation, Validation, Writing – review & editing. PC: Investigation, Validation, Writing – review & editing. YZ: Investigation, Software, Validation, Writing – review & editing. HD: Supervision, Validation, Writing – review & editing. KD: Data curation, Investigation, Supervision, Writing – review & editing. XY: Conceptualization, Writing – review & editing. HL: Conceptualization, Funding acquisition, Project administration, Resources, Supervision, Writing – review & editing. CH: Conceptualization, Methodology, Writing – review & editing.
